# Prevalence of Antimicrobial Resistant Bacteria from Conjunctival Flora in an Eye Infection Prone Breed (Saint Bernard)

**DOI:** 10.3390/molecules26082219

**Published:** 2021-04-12

**Authors:** George Cosmin Nadăș, Cristiana Ștefania Novac, Ioana Adriana Matei, Cosmina Maria Bouari, Zoltan Miklos Gal, Octavia Maria Tamas-Krumpe, Adrian Maximilian Macri, Nicodim Iosif Fiț

**Affiliations:** 1 Department of Microbiology, Faculty of Veterinary Medicine, University of Agricultural Sciences and Veterinary Medicine, 400372 Cluj-Napoca, Romania; gnadas@usamvcluj.ro (G.C.N.); cristiana.novac@usamvcluj.ro (C.Ș.N.); cosmina.bouari@usamvcluj.ro (C.M.B.); nfit@usamvcluj.ro (N.I.F.); 2 Department of Reproduction, Obstetrics and Pathology of Reproduction, Faculty of Veterinary Medicine, University of Agricultural Sciences and Veterinary Medicine, 400372 Cluj-Napoca, Romania; zoltan.gal@usamvcluj.ro; 3 Department of Physiology, Faculty of Veterinary Medicine, University of Agricultural Sciences and Veterinary Medicine, 400372 Cluj-Napoca, Romania; octavia.tamas@usamvcluj.ro; 4 Department of Animal Nutrition, Faculty of Veterinary Medicine, University of Agricultural Sciences and Veterinary Medicine, 400372 Cluj-Napoca, Romania; adrian.macri@usamvcluj.ro

**Keywords:** AMR, MDR, ocular flora, *Pseudomonas*, Saint Bernard, *Staphylococcus*

## Abstract

The conjunctival bacterial resident and opportunistic flora of dogs may represent a major source of dissemination of pathogens throughout the environment or to other animals and humans. Nevertheless, contamination with bacteria from external sources is common. In this context, the study of the antimicrobial resistance (AMR) pattern may represent an indicator of multidrug resistant (MDR) strains exchange. The present study was focused on a single predisposed breed—Saint Bernard. The evaluated animals were healthy, but about half had a history of ocular disease/treatment. The swabs collected from conjunctival sacs were evaluated by conventional microbiological cultivation and antimicrobial susceptibility testing (AST). The most prevalent Gram-positive was *Staphylococcus* spp.; regardless of the history, while Gram-negative was *Pseudomonas* spp.; exclusively from dogs with a history of ocular disease/treatment. Other identified genera were represented by *Bacillus*, *Streptococcus*, *Trueperella*, *Aeromonas* and *Neisseria*. The obtained results suggest a possible association between the presence of mixed flora and a history of ocular disease/treatment. A high AMR was generally observed (90%) in all isolates, especially for kanamycin, doxycycline, chloramphenicol and penicillin. MDR was recorded in *Staphylococcus* spp. and *Pseudomonas* spp. This result together with a well-known zoonotic potential may suggest an exchange of these strains within animal human populations and the environment.

## 1. Introduction

The conjunctival bacterial resident population is an important component of the local defense, and it helps to prevent colonization by the overgrowth of potentially pathogenic agents [1,2]. The predominant saprophytic organisms of the dog eye are Gram-positive bacteria, while Gram-negative bacteria presence is rather uncommon. The most common reported Gram-positive bacteria are the members of *Staphylococcus* spp.; *Bacillus* spp.; *Streptococcus* spp. and *Trueperella* spp. Gram-negative bacteria such as *Escherichia coli*, *Pseudomonas* spp.; *Neisseria* spp. and *Klebsiella* spp. are also frequently reported [3,4,5,6,7]. However, their saprophytic or opportunistic nature is not well-documented or stated. The structure of saprophytic flora may be slightly influenced by different internal factors (age, sex and breed) or external factors (season, geography and climate) [5,8]. However, some of these factors may not have a statistically significant influence [5].

The knowledge regarding normal ocular flora structure is of major importance, since the same organisms are the predominant agents recovered from ocular infection [3,5]. Opportunistic bacteria such as: *Staphylococcus* spp.; *Streptococcus* spp.; *Pseudomonas* spp.; *E. coli*, *Trueperella* spp. and *Bacillus cereus* may become potential pathogens if tissue damage occurs or if the organism’s resistance to infection is decreased [9]. Withal, the destruction of the normal ocular flora by long-term use of topical antimicrobials can result in overgrowth of pathogenic bacteria, yeasts or fungi [9]. In addition, conformational abnormalities such as entropion or ectropion may contribute to secondary opportunistic infections [8]. The entropion may represent a predisposing factor, since it causes conjunctiva irritation or even trauma with ulceration of the cornea [10]. The ectropion results in high exposure of the conjunctiva, potentially able to cause conjunctivitis and keratitis [10] For instance, Wang et al. [5] evaluated the prevalence of positive bacterial culture on 13 dog breeds, showing a higher prevalence in Pug, Pekingese and Schnauzer. Despite the increased exposure of Pug’s conjunctiva (exophthalmic breed), which may explain the high prevalence of this breed, the small number of tested animals was not sufficient to establish the presence of a statistically significant difference [5].

Alongside other breeds with genetic predilections for conformational abnormalities, the Saint Bernard is highly predisposed to both entropion and ectropion, having a so-called “diamond eye” [10,11]. However, to the best of our knowledge, there are no studies regarding the influence of these abnormalities on the resident or opportunistic flora or on Saint Bernard predisposition to eye infections.

Ocular disease associated with bacterial agents, either primarily or secondarily, represents a common problem in small animal practice, especially from the treatment point of view [8]. The most frequently used formulas are mixtures between anti-inflammatory agents with two or even three antibiotics that cover a large spectrum of agents. Commonly used formulas are combinations between neomycin, polymyxin B and gramicidin (solution); or neomycin, polymyxin B and bacitracin (ointment), with efficacy on both the Gram-negative bacteria (neomycin and polymyxin B) and Gram-positive bacteria (bacitracin and, gramicidin) reviewed by reference [12]. Other frequently used antimicrobial agents are aminoglycosides: 3% tobramycin and 0.3% gentamicin, fluoroquinolones: 0.3% ofloxacin, 0.3% ciprofloxacin (solution and ointment), 0.5% levofloxacin, 0.5% moxifloxacin, 0.3% gatifloxacin and 0.6% besifloxacin and chloramphenicol (1% ointment) reviewed by reference [12]. Due to the long-term and high-frequency use of these agents, different resident or opportunistic bacteria were reported in the last years as being resistant to all previously mentioned drugs [13,14,15,16]. Nevertheless, the resistance of bacteria to certain antimicrobials not necessarily used in topic therapy may indicate either an influence of systemic therapy (used for treating other infections) or a circulation of resistant bacteria within the human-animal-environment interface. In this context, our aims were to evaluate: (i) the bacteria diversity and their frequency of in Saint Bernard eyes, (ii) the possible influence of individual and external factors on bacteria diversity, (iii) the antimicrobial resistance (AMR) to commonly used broad-spectrum drugs of isolated bacteria and (iv) to discuss the possible dissemination of resistant bacteria within the human-animal-environment interface.

## 2. Results

The population included in this study was represented by 28 females (56%) and 22 males (44%) aged one to eight years (mean age 4.2). A total number of 26 dogs (52%) had clinical ocular infections and were treated with topical antibiotic (Mibazon^®^: 2.5-g tetracycline, 1.2-g erythromycin, 1.2-g neomycin sulphate and 0.06-g prednisolone/100 g ointment) in the last year. In 41 (82%, CI 95%: 68.56–91.42) cases, bacteria were isolated. The predominant identified flora was Gram-positive bacteria (100%), Gram-negative only being isolated in eight cases (19.51%, CI 95%: 8.82–34.87) (Table 1 and Table 2). In twenty cases (48.78%, CI 95%: 32.88–64.87), a pure culture was obtained. In the remaining cases, the associations of two (*n* = 16, 39.02%, CI 95%: 24.2–55.5), three (*n* = 4, 9.76% CI 95%: 2.72–23.13) or four (*n* = 1, 2.44%, CI 95%: 0.06–12.86) genera were observed (Table 2). The most frequent identified genus was *Staphylococcus*, followed by *Micrococcus* and *Trueperella* (Table 1). Among Gram-positive genera, *Streptococcus* and *Bacillus* were less frequently isolated. Gram-negative-identified genera were *Pseudomonas*, *Aeromonas* and *Neisseria* (Table 1).

Age and sex seem to have no influence on the presence and prevalence of the different identified genera. Instead, the history of clinical infections/treatment seems to have an impact on the flora structure. The prevalence of Gram-negative flora was significantly higher from a statistical point of view in treated dogs compared to untreated dogs (30.43% vs. 5.56%, *p* = 0.04). *Streptococcus*, *Neisseria* and *Pseudomonas* genera were exclusively isolated from dogs with antibiotic treatment history (*p* = 0.03, 0.19 and 0.06, respectively). However, in the case of *Pseudomonas* and *Neisseria*, the difference was not statistically significant due to the small number of positive samples.

The efficient antibiotics were ciprofloxacin, marbofloxacin and ofloxacin with 97.5%, 80.5% and 82.9%, respectively, susceptible isolates, followed by gentamicin, amoxicillin and clavulanic acid (AMC) both with 78.1% and tobramycin with 75.6% susceptible isolates. A moderate frequency of susceptible isolates was observed for florfenicol with 68.3%, cephalexin 63.4%, neomycin 61% and tetracycline with 58.5%. Less efficient can be considered doxycycline 34.2% and penicillin and kanamycin, both with 22%, and chloramphenicol with 17.1% susceptible isolates. All isolates were resistant to bacitracin. A medium percent of the isolates was resistant to two-to-four antibiotics (to two: 9.8%, three: 4.9% and four: 17.1%). The resistance to several drugs was recorded in 24.4% of the isolates for five antibiotics, 4.9% for six and 7.3% for seven. Resistance for at least half of the tested antimicrobials were in 4.9% of isolates for eight antibiotics, 9.8% for nine, 2.4% for 10 and 11, 4.9% for 12 and 13 and 2.4% for 15 antibiotics. The resistance to multiple drugs (more than seven) was recorded for only several genera: in 12 *Staphylococcus* spp. strains, four *Pseudomonas* spp. strains, three *Micrococcus* spp. and one *Streptococcus* spp. strain. Within *Staphylococcus* spp.; the majority of isolates were resistant to bacitracin, chloramphenicol, kanamycin, doxycycline and penicillin, while only a few isolates were resistant to ciprofloxacin, marbofloxacin and AMC (Table 3). *Pseudomonas* spp. was highly resistant, being susceptible to ciprofloxacin, marbofloxacin and ofloxacin (Table 3). A single *Staphylococcus* spp. strain showed resistance to all tested antibiotics.

The ocular flora structure seems to be the only factor to have an influence on the frequency of resistant bacteria to multiple antimicrobials. The percent of Gram-negative bacteria resistant to more than seven antimicrobials was significantly higher, *p* = 0.01 (100% *Pseudomonas* spp. vs. 60% *Micrococcus* spp. or 31.6% *Staphylococcus* spp.-resistant strains).

## 3. Discussion

Considering the common use of ophthalmic therapy in veterinary practice and the significance of AMR in the past decades, the study of ocular flora/pathogens and their AMR pattern is of major importance. In addition, the present study focused on one predisposed breed, highlighting the importance and influence of ocular disease and treatment history on normal ocular flora and the AMR pattern. Although the homogenous population with a common origin (same kennel) may be an advantage as the influencing external factors are similar, this aspect can be also a limitation of the study, since it is not clear how it will be applied to the general dog population.

In the present study, more than half of the Saint Bernard’s included had ocular disease and treatment history. This is in agreement with the literature and well-known predisposition of this breed. This predisposition was associated with the length of the eyelid and the firmness of the lateral canthus, resulting in an overlong lid with little internal support that will naturally deform, while a slack lateral canthus allows the palpebral fissure to droop and distort [10]. According to the present observations, the Gram-positive flora, including *Staphylococcus*, *Trueperella* and *Micrococcus* genera, seem to predominate in both healthy and treated dogs, while Gram-negative microorganisms (i.e., *Pseudomonas* spp.; *Aeromonas* spp. and *Neisseria*) were more frequently detected in individuals with disease and treatment history.

Despite the fact that members of the genus *Staphylococcus* are commonly isolated from ocular microbiota, being considered commensal on mucosal surfaces, many species may also act as opportunistic pathogens and cause eye-related infections [17,18,19]. Among different species of the genus, the most predominantly involved in ocular diseases seems to be *S. intermedius*, followed by *S. aureus* and less coagulase negative staphylococci [18]. Although the second-most detected genus in this study was *Trueperella*, other studies have suggested a minor involvement in the pathogenesis of ocular diseases [19,20]. Conversely, the genus *Micrococcus* seems to be detected from both normal and pathogenic flora [20,21,22].

Gram-negative rods are part of the normal microbiota but in a lower proportion (~10%) compared to Gram-positive ones [20,23,24]. However, the Gram-negative flora presence is important, since they usually may act as opportunistic bacteria. The most reported saprophytic flora is represented by the genera *Moraxella*, *Acinetobacter* and *Neisseria* [24,25]. In addition to these, *Pseudomonas aeruginosa*, *Pasteurella* spp.; *Escherichia coli*, *Enterobacter* spp.; *Klebsiella* spp. and *Proteus* spp. are also isolated from dogs with ocular disease history [19,24,26]. The difference in the frequency of Gram-negative rods (i.e., *Pseudomonas* spp.; *Aeromonas* spp. and *Neisseria* spp.) between the healthy and treated dogs observed in this study sustain the involvement of Gram-negative rods as opportunists (*p* = 0.04). Based on this, it can be assumed that prior treatment may help Gram-negative overgrowth. Although, in the present study, the prior treatment consisted of a triple combination (tetracycline, erythromycin and neomycin), the impact on Gram-negative overgrowth may be the same regardless of the antimicrobials used. In this case, Gram-negative bacteria (including resistant strains) that can act more often as opportunistic pathogens [19,24,26] may circulate within the human-animal-environment interface, representing a public health risk.

The ocular components and, mainly, the conjunctive mucous membrane are constantly exposed to contamination with bacterial flora from the environment. These microbes may have developed a resistance to antibiotics, and, if they attach and colonize, ocular disease may develop. Furthermore, these antimicrobial-resistant microbes may be disseminated in the environment and to other susceptible hosts, including man. The dissemination of resistant bacteria within human-animal-environment interface has been extensively discussed and studied. White [27], in a review, discussed how the interaction between microecological systems in different hosts (including humans) and the environment may influence the transfer of resistant bacteria and their resistance genes [27]. Moreover, it highlights the pronounced capacity for the acquisition and transfer of resistance genes of commensals in ecosystems [27]. The transfer routes discussed in this review, by meat products or via other routes such as water and food plants [27], complement the direct transfer mechanism of zooanthroponotic agents within animal-human populations. Based on these considerations, the AMR pattern of the ocular flora may indicate a public health risk. In the present study, the most resistant isolates to antimicrobials were represented by *Pseudomonas* and *Staphylococcus* genera members.

The *Pseudomonas* genus includes *P. aeruginosa*, a well-known MDR zoonotic pathogen. According to the latest ECDC (European Center for Disease Prevention and Control) report, a high percentage of *P. aeruginosa* isolated from human blood cultures presented a resistance to several antimicrobial groups, including aminoglycosides, fluoroquinolones, carbapenems, etc. [28]. The zooanthroponotic potential of this species was previously reported [29,30]. In addition, the circulation of a resistant strain within pet owner-environment interfaces was also highlighted [29]. Unfortunately, in the veterinary sector, the surveillance of AMR is scarce. However, there are several reports on AMR *P. aeruginosa* isolated from domestic carnivores. For instance, Rubin et al. [31] isolated highly resistant *P. aeruginosa* strains from canine otitis and pyodermitis. A high percentage of resistance was recorded for β-lactams (ampicillin, cefoxitin, cefpodoxime, cephalothin, cefazolin, AMC and ceftiofur); for quinolones and fluoroquinolones (naladixic acid and orbifloxacin); to aminoglycosides (kanamycin and streptomycin) and to chloramphenicol and tetracycline [31]. Similarly, Li et al. [32] detected *Pseudomonas* spp. in clinical samples from domestic carnivores resistant to AMC, cefoxitin, ampicillin, cefotaxime and cefuroxime [32]. *Pseudomonas aeruginosa* strains isolated from ocular disease showed 100% susceptibility only to imipenem and a high resistance to enrofloxacin (75%) [16]. All these observations are in agreement with the results reported here, showing a high resistance to most antimicrobial drugs (β-lactams, aminoglycosides, cephalosporines, chloramphenicol and florfenicol), except for marbofloxacin. Overall, quinolones seem to have a better effectiveness against *P. aeruginosa*—specifically, marbofloxacin, norfloxacin, gatifloxacin, difloxacin, enrofloxacin, levofloxacin and ciprofloxacin [16,31] in the present study.

The *Staphylococcus* genus includes species with zoonotic potential (e.g., *S. aureus* and *S. pseudintermedius*) and previously reported AMR. Although, according to the latest official reports of the ECDC, the MRSA (multidrug resistant *Staphylococcus aureus*) frequency is decreasing, it remains an important pathogen due to the combined resistance to other antimicrobial groups [28]. Both *S. aureus* and *S. pseudintermedius* (including methicillin-resistant strains) have shown a zoonotic potential, being especially transmitted from dogs to humans [33,34,35,36]. Staphylococci, especially coagulase-negative species but, also, coagulase-positive ones (including *S. aureus*, *S. intermedius* and *S. pseudintermedius*) are commensals of the ocular surface with the potential to become opportunistic pathogens [18]. In the present study, staphylococci were isolated from both healthy dogs and dogs with ocular disease/treatment history. The majority of isolated strains presented are resistance to bacitracin, chloramphenicol, penicillin, kanamycin and doxycycline (Table 3). A variable frequency (20–40%) of strains resistant to quinolones and β-lactams was recorded. In addition, 20 strains presented MDR. Similar results were obtained by Varges et al. [18], where a high resistance to penicillin and a moderate one to cephalexin and AMC was also observed for *Staphylococcus* spp. ocular isolates. Herein, a concerning percentage of MDR strains was reported [18]. Among different species of staphylococci, *S. intermedius* seems to have a limited resistance [13], whereas *S. pseudintermedius* seems to present a high resistance to different antimicrobial classes [15]. In this study, more than half of *S. pseudintermedius* strains were resistant to tetracycline, penicillin, sulfamethoxazole/trimethoprim, erythromycin and gentamycin [15]. Notably, a relatively high percentage (34%) of these strains also presented resistance to oxacillin, suggesting a high prevalence of MRSP (multidrug resistant *Staphylococcus pseudintermedius*) [15]. Accordingly, MRS strains, including *S. epidermidis*, *S. pseudintermedius* and *S. aureus*, were isolated from canine corneal samples [37]. Although in the present study, the resistance to methicillin was not evaluated, the detection of MRS strains in other studies is of major importance, since the resistance to methicillin seems to be associated with a cross-resistance to other antimicrobial classes, as it was shown for some fluoroquinolones (ciprofloxacin and ofloxacin) [14].

The overall antimicrobial resistance patterns observed in the present study, although evaluated by the disk diffusion test, are comparable with the literature. This study included broad-spectrum antimicrobials used in both veterinary and human medicine, aiming to highlight the possible circulation of these bacteria within different hosts and the associated risk, altogether raising high concerns regarding the possible circulation of resistant strains in animals. Since most of detected bacteria have a zoonotic potential, they may also be transmitted to humans, either directly or through environmental contamination, representing a public threat.

## 4. Materials and Methods

### 4.1. Animal and Specimen Collection

A total of fifty Saint Bernard dogs from a private kennel with no clinical signs of conjunctivitis were evaluated during April–September 2018 regarding the presence, type of microbial flora and susceptibility to antimicrobial agents. The dogs were healthy at the beginning of the study, their health status confirmed by the owner and the result of a general examination.

The samples were collected using sterile cotton swabs, dry and premoistened in sterile saline. The sampling involved separate eye collection, and no anesthetics were applied. The swabs were used to swipe and roll the lower conjunctival sac directed to the nictitating membrane, avoid touching neighboring areas, such as eyelid margins, genes or cornea, that may contaminate the sample. Sterile Amies medium tubes for transportation were used for transfer. The samples were transported the same day to the microbiology laboratory.

### 4.2. Bacteriological Examination

In the microbiology laboratory, each swab was used to inoculate Columbia agar with 5% sheep blood and MacConkey agar using the T-streak technique (three sections) and incubated under aerobic conditions at 36 ± 2 °C for 18–24 h. The swabs were then placed into a thioglycolate broth, used as a supplementary enrichment culture and again incubated under aerobic conditions at 36 ± 2 °C for 18–24 h. The next day, samples from the broth were used to inoculate Columbia agar with 5% sheep blood and incubated in aerobic conditions at 36 ± 2 °C for 18–24 h. The identification of bacteria at the genus level was performed by microscopy (Gram staining) cultural examination, followed by biochemical characters (Supplementary Table S1). Randomly selected strains (approx. 10%) were identified using the Vitek^®^ 2 compact 15 system (bioMérieux, Marcy l’Etoile, France) confirming the genus level identification by the protocol used in this study (Supplementary Table S1).

### 4.3. In Vitro Susceptibility Testing

The evaluation of susceptibility to antimicrobials was performed using the disk diffusion method. Mueller–Hinton agar plates were flooded with 1 mL from the suspension of isolated colonies in broth at a 0.5 McFarland density scale using Densicheck^TM^ Plus (bioMérieux, Marcy l’Etoile, France). The antibiotics were represented by AMC 20/10 µg, Bacitracin 0.04UI, chloramphenicol 30 µg, cephalexin 30 µg, ciprofloxacin 5 µg, doxycycline 30 µg, Florfenicol 30 µg, gentamicin 10 µg, kanamycin 30 µg, marbofloxacin 5 µg, neomycin 30 µg, ofloxacin 5 µg, penicillin 10UI, tetracycline 30 µg and tobramycin 10 µg. Zone diameter breakpoints in mm were considered as follows: AMC 13, bacitracin 8, chloramphenicol 12, cephalexin 17, ciprofloxacin 15, doxycycline 18, florfenicol 18, gentamicin 12, kanamycin 13, marbofloxacin 14, neomycin 14, ofloxacin 12, penicillin 24, tetracycline 17 and tobramycin 12, based on the lowest available EUCAST (European Committee on Antimicrobial Susceptibility Testing) and CLSI (Clinical and Laboratory Standards Institute) breakpoints for a certain genus (Supplementary Table S1).

### 4.4. Statistical Analysis

Statistical analysis was performed using Epi Info^TM^7 (CDC, Atlanta, GA, USA) software. The pathogen prevalence and their susceptibility to antibiotics and the prevalence of bacteria resistant to multiple antimicrobials (>7) were analyzed with the chi-square independence test. Statistical significance was defined as *p* < 0.05.

## 5. Conclusions

As it was observed in the present study and similar studies in the field, staphylococci represent major group of commensals that may act as opportunistic pathogens, being also frequently isolated from dogs with ocular disease history. Similarly, Gram-negative bacteria, including *Pseudomonas* spp.; are recognized as both commensals and opportunists. Herein, a higher frequency of mixed Gram-positive and -negative flora in dogs with ocular disease/treatment history were observed. Due to the small sample number included in the present study, it is not clear whether Gram-negative bacteria act more often as opportunistic or if the local treatment imbalanced the microflora structure; therefore, this aspect should be further investigated. Of major concern were the common AMR and MDR observed in both the most prevalent Gram-positive (i.e., *Staphylococcus* spp.) and Gram-negative (i.e., *Pseudomonas* spp.) members. The presence of MDR strains in dogs may represent an important threat from the One Health point of view, due to the likely dissemination of these strains within animal and human populations and the environment.

## Figures and Tables

**Table 1:** The prevalence of bacterial genera identified in ocular secretion in Saint Bernard dogs.

Pathogen/Genus	Frequency (*n*/%)
*Staphylococcus*	38/92.68
*Micrococcus*	7/17.07
*Trueperella*	9/13.04
*Streptococcus*	5/12.2
*Pseudomonas*	4/9.76
*Aeromonas*	2/4.88
*Neisseria*	2/4.88
*Bacillus*	2/2.90

**Table 2:** The bacterial associations identified in ocular secretion in Saint Bernard dogs.

Bacteria Detected	Number of Cases
*Staphylococcus*, *Trueperella*	5
*Staphylococcus*, *Micrococcus*	3
*Staphylococcus*, *Streptococcus*	1
*Staphylococcus*, *Trueperella*, *Micrococcus*	1
*Staphylococcus*, *Trueperella*, *Bacillus*	1
*Staphylococcus*, *Micrococcus*, *Streptococcus*	1
*Streptococcus*, *Micrococcus*	1
*Streptococcus*, *Bacillus*	1
*Staphylococcus*, *Pseudomonas*^1^	4
*Staphylococcus*, *Aeromonas*^1^	1
*Staphylococcus*, *Neisseria*^1^	1
*Staphylococcus*, *Trueperella*, *Neisseria*^1^	1
*Staphylococcus*, *Trueperella*, *Streptococcus*, *Aeromonas*^1^	1

^1^ Association between Gram-positive and -negative bacteria.

**Table 3:** The frequency of resistant bacteria isolates in Saint Bernard eyes to the tested antimicrobials.

	Bacterial Genera							
	*Staphylococcus*	*Streptococcus*	*Trueperella*	*Bacillus*	*Micrococcus*	*Aeromonas*	*Pseudomonas*	*Neisseria*
AMC	6/38	1/5	0/9	0/2	1/7	0/2	4/4	0/2
BAC	38/38	5/5	9/9	2/2	7/7	2/2	4/4	2/2
CAP	33/38	4/5	7/9	1/2	5/7	2/2	4/4	2/2
CEX	11/38	2/5	2/9	0/2	6/7	0/2	4/4	0/2
CPF	1/38	0/5	0/9	0/2	0/7	0/2	0/4	0/2
DOX	26/38	3/5	7/9	1/2	5/7	2/2	4/4	2/2
FLO	13/38	0/5	1/9	0/2	2/7	0/2	4/4	0/2
GEN	8/38	1/5	0/9	0/2	1/7	0/2	4/4	0/2
KAN	31/38	4/5	8/9	1/2	4/7	2/2	3/4	2/2
MBX	6/38	0/5	0/9	0/2	2/7	0/2	0/4	0/2
NEO	15/38	1/5	1/9	0/2	2/7	0/2	4/4	1/2
OFX	7/38	0/5	1/9	0/2	0/7	0/2	2/4	0/2
PCN	28/38	3/5	6/9	1/2	6/7	1/2	4/4	1/2
TET	15/38	1/5	1/9	0/2	3/7	0/2	4/4	0/2
TOB	9/38	1/5	1/9	0/2	1/7	0/2	3/4	0/2

Legend: AMC—Amoxicillin and clavulanic acid, BAC—Bacitracin, CAP—chloramphenicol, CEX—cephalexin, CPF—Ciprofloxacin, DOX—doxycycline, FLO—Florfenicol, GEN—Gentamicin, KAN—kanamycin, MBX—Marbofloxacin, NEO—Neomycin, OFX—Ofloxacin, PCN—penicillin, TET—Tetracycline and TOB—Tobramycin.

## Data Availability

No new data were created or analyzed in this study. Data sharing is not applicable to this article.

## Supplementary Materials

Table S1: Supplementary data of bacterial genera identification criteria and breakpoints establishing criteria.

## Sample Availability

Not available.
