# Supplementary material for: Prevalence of Antimicrobial Resistant Bacteria from Conjunctival Flora in an Eye Infection Prone Breed (Saint Bernard)
Source: Molecules. 2021 Apr 12;26(8):2219. doi: 10.3390/molecules26082219 (PMC8070223; doi:10.3390/molecules26082219)
Supplement: Supplementary file 1 [file molecules-26-02219-s001.pdf]

Genus*	**Gram**	**Colonies**	**Cell morphology**	**Respiratory type**	**Catalase**	**Oxidase**	**Hemolys.is**	**Indole**	**H_2S**	**Urea hydrolysis**
Staphylococcus spp.	+	Opaque, pigmented	Cocci, pairs and clusters	Facultative anaerobes	+	-	+/-	NA	NA	NA
Streptococcus spp.	+	Translucent, pinpoint	Spherical/ovoid, pairs or chains	Facultative anaerobes	-	-	+	NA	NA	NA
Micrococcus spp.	+	Yellow colonies	Spherical, pairs, tetrads, clusters	Aerobes	+	+	-	NA	NA	NA
Trueperella spp.	+	Convex and semi opaque, matte surface	Straight or curved rods, pairs, V formation	Facultative anaerobes	+	-	+/-	NA	NA	NA
Bacillus spp.	+	Large, flat, irregular	Rods, pairs or chains	Aerobic/facultative anaerobes	+	-	+	NA	NA	NA
Aeromonas spp.	-	Medium, translucent, convex	Rods, rounded ends, single, pairs or short chains	Facultative anaerobes	+	+	+	+	+	-
Pseudomonas spp.	-	Diffusible green pigment	Rods	Aerobes	+	+	+	-	-	-
Neisseria spp.	-	Yellow carotenoid pigment	Cocci, pairs	Aerobes	+	+	-	-	-	-

* 5 *Staphylococcus pseudintermedius, 1 Streptococcus pyogenes, 2 Micrococcus luteus, 2 Trueperella pyogenes, 1 Bacillus cereus, 1 Aeromonas sobria, 2 Pseudomonas aeruginosa* and 1 *Neisseria flava* were identified were identified using Vitek® 2 Compact 15 system (bioMérieux, Marcy l'Etoile, France).

	AMC	CEX	CAP	DOX	FLO	KAN	MBX	PNC	TOB	GEN	NEO	TET	BAC	CPF	OFX
Staphylococcus	19*	17*	12**	20*	NE	19*	14**	28**	22*	12**	14*	17*	8*	15*	14**
Trueperella	13*	NE	NE	NE	NE	NE	NE	29*	NE	NE	NE	24*	NE	25*	NE
Micrococcus	13*	NE	NE	NE	NE	NE	NE	NE	NE	NE	NE	NE	NE	NE	NE
Streptococcus	13*	17*	17**	18**	18**	NE	14**	24**	NE	NE	NE	18*	NE	NE	NE
Pseudomonas	13*	17**	NE	NE	NE	NE	16**	NE	12**	12**	NE	NE	NE	18**	12**
Bacillus	13*	NE	NE	NE	NE	NE	NE	NE	NE	NE	NE	NE	NE	23*	NE
Aeromonas	13*	NE	NE	30**	33**	NE	NE	NE	NE	24**	NE	28**	NE	24*	NE
Neisseria	13*	23**	19**	NE	NE	NE	NE	26**	NE	NE	NE	30**	NE	32**	NE
Considered value	13	17	12	18	18	13***	14	24	12	12	14	17	8	15	12

*EUCAST; **CLSI (VET+M100); *** CLSI (Enterobacteriaceae); AMC – Amoxicillin and clavulanic acid, Bacitracin – BAC, CAP – chloramphenicol, CEX – cephalexin, CPF – Ciprofloxacin, DOX – doxycycline, FLO – Florfenicol, GEN – Gentamicin, KAN – kanamycin, MBX – Marbofloxacin, NEO – Neomycin, OFX – Ofloxacin, PCN – penicillin, TET – Tetracycline, TOB – Tobramycin.
